# Provision of trauma care in asymmetric warfare: a conceptual framework to support the decision to implement frontline care services

**DOI:** 10.1186/s13031-022-00490-w

**Published:** 2022-10-29

**Authors:** F. Salio, A. Pirisi, E. Bruni, M. Court, K. Peleg, S. Reaiche, A. Redmond, E. Weinstein, I. Hubloue, F. Della Corte, L. Ragazzoni

**Affiliations:** 1 World Health Organization (WHO), Geneva, Switzerland; 2 CRIMEDIM – Center for Research and Training in Disaster Medicine, Vrije Universiteit Brussel (VUB), Humanitarian Aid and Global Health Università del Piemonte Orientale Via Lanino, 1, 28100 Novara, Italy; 3 Beth Israel Deaconess Medical Center, Harvard Medical School, Boston, USA; 4 Department of Emergency and Disaster Management, Tel Aviv University, Tel Aviv, Israel; 5 Geneva Call, Geneva, Switzerland; 6 Humanitarian and Conflict Response Institute, University of Manchester, Manchester, UK; 7 Research Group on Emergency and Disaster Medicine, Vrije Universiteit Brussel, Brussels, Belgium; 8 World Health Organization, Ukraine Country Office, Kyiv, Ukraine

**Keywords:** Asymmetric warfare, Emergency and trauma care, Pre-hospital care, Trauma Stabilization Points

## Abstract

**Introduction:**

The emerging trends of asymmetric and urban warfare call for a revision of the needs and the way in which frontline trauma care is provided to affected population. However, there is no consensus on the process to decide when and how to provide such lifesaving interventions in form of Trauma Stabilization Point (TSP).

**Methods:**

A three-step Delphi method was used to establish consensus. A focus group discussion was convened to propose a framework and develop the list of twenty-one (21) statements for validation of a group of experts.

**Results:**

A panel of twenty-eight (28) experts reviewed the statements and participated to both first and second rounds. Comments and recommendations provided by the FGD and during round 1 were used to analyze the findings of the study. The proposed framework includes five main categories identified as interconnected components that facilitate the decision to implement or not the TSP. A total of sixteen (16) elements distributed across the five categories have been considered as being able to guide the decision to utilize such capability in high-risk security and resource constrained settings.

**Conclusion:**

The TSP has the potential to prevent death and disability. The proposed framework and categories add a structure to the decision-making process and represents an important step to support emergency and trauma care planning and implementation efforts.

## Introduction

The emerging trends of asymmetric and urban warfare open a needed debate that was predicted to occur once intrastate, non-international armed conflicts began to dominate over interstate or cross-borders wars during the post-Cold War era which have today all but disappeared [1, 2]**.** Asymmetric conflict brought tension between humanity and the demand of military operations and, when respect by one-part fades, mutual disrespect for the adversary and the law of armed conflict increases [3]. While emphasizing the role of each party in providing frontline care to the wounded, it is argued that asymmetry in war expand the range of permissible civilian targets without each side incurring charges of terrorism or disproportionate harm [4].

Military research and the analysis of the way in which trauma care is executed on the frontline have significantly contributed to improvements in the clinical outcomes of injured soldiers and redefined the trauma paradigm towards improving efficiency. By moving medical capabilities as close as possible to the point of injury (POI), better tactical pre-hospital care and reducing the time from POI to the casualty arriving at a medical facility, death rates on the battlefield have decreased significantly [5, 6].

The extent to which civilian and military trauma care and innovation have been of mutually reinforcing benefit is reflected in the successful adoption of systems and processes into the civilian trauma settings. However, most research on trauma care in conflict settings has been done in the context of symmetric warfare [7]. Nowadays, this is challenged by external factors such as nonlinear battlefields, the principle of distinction and the rising need for civilian actors to provide and adjust treatment capacity to the acute increase in demand and to sustain its functionality for prolonged periods [8]. Considering the above and the direct correlation between proximity and effectiveness of medical aid operations, the concept of the Trauma Stabilization Point (TSP) is introduced.

The TSP is proposed as the first site of care staffed by trained medical personnel. Its primary function is to provide far-forward emergency resuscitation and stabilization and must be capable of functioning in resource-constrained environments [9].

There is no consensus on the process to decide when and how to provide frontline trauma care services in the context of asymmetric warfare. The time is right to bring varied experiences and experts together to agree on a commonly accepted framework to support this process and better allow future dissemination of best practices.

This study aims to describe the development of a conceptual framework to support the decision to implement frontline care services by using expert consensus process. This framework serves as a necessary first step to foster critical debate for health care decision-making to facilitate the decision on how to implement tactical pre-hospital care in complicated and demanding intrastate conflicts.

## Methods

### Study design

The Delphi method has been selected to make effective use of informed intuitive judgement and derives from personal expectations from individuals rather than predictions from well-established theory. A convergence of opinion has been observed in the majority of cases where the Delphi approach has been used. It provides anonymity for respondents, the possibility to review and assess the comments and feedback provided by the other Delphi panelists, a controlled feedback process, and the suitability of a variety of statistical analysis techniques to interpret the data [10]. A three-step Delphi method was used to establish consensus and, considering the limited evidence, available in the literature, a focus group discussion was set up to develop the list of statements to be submitted to the group of experts.

### Panel selection

**Fig. 1:**
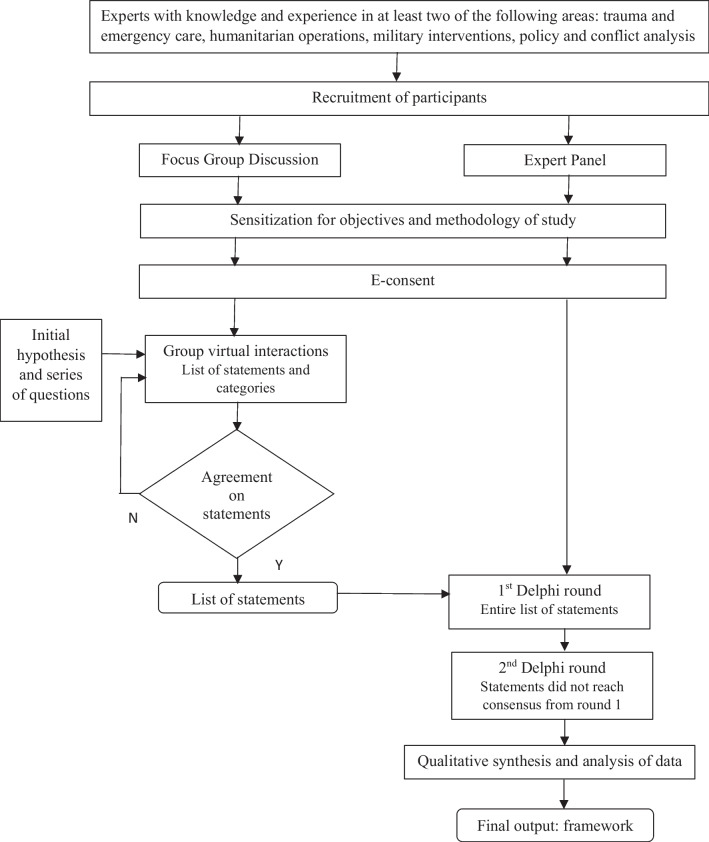
Methodology in the Delphi consensus study

The criteria used to guide the selection of the Focus Group Discussion (FGD) and Delphi experts included consideration of individuals who were highly trained and competent within their specialized area of knowledge and expertise who might potentially utilize the outcomes of the study. Therefore, experts were chosen based on sector expertise and experience in trauma and emergency care, humanitarian operations, military interventions, policy and conflict analysis with a willingness to revise their initial or previous judgments for the purpose of reaching or attaining consensus. Twenty-eight experts out of the thirty-two contacted provided consent and agreed to participate while ten experts formed part of the FGD (Fig. 1).

### Focus group discussions

A brief document containing a clear explanation of the objective of the study and specific instructions for member participation was circulated by email to all ten members of the FGD. All the members were familiar with the TSP concept as were involved or exposed to it during its first iteration in Mosul, Iraq in 2016–17 and the subsequent fighting in Raqqa, Syria.

The group was tasked with (1) the review and discussion related to the problem, its magnitude and the agreement on the need of such intervention; (2) the creation of a list of statements representing key considerations to be made for the implementation of the TSP; (3) the validation of the statements for the Delphi study.

No research is available pertaining to the necessary process to facilitate the decision to implement the TSP. The initial hypothesis was guided by the fact that civilian medical systems and personnel are not set and trained on how to operate clinically in semi-permissive and non-permissive environments. Thus, a series of questions focused on the need for such approach, its role and scope, and what influence its implementation have facilitated the interaction among the FGD members.

The process ended with the definition of a list of 21 statements grouped into categories representing the structure of the framework. FGD members were encouraged to discuss the statements until agreement was reached. The draft document containing the list of statements divided in 5 categories was circulated by email to all 10 members of the FGD for confirmation while the opportunity to provide additional comments and recommendations was given and feedback recorded.

### Delphi round 1

A brief document containing a clear explanation of the objective of the study and specific instructions for member participation was circulated by email to all twenty-eight panel members. Experts who agreed to partake in the study were sent an email invitation to create a username and password on the Stat59 online platform (Stat59 Services Ltd, Edmonton, AB, Canada) where the surveys were managed. Seven point linear numeric scales were used, and each expert was requested to assign a point value ranging from 1 (strongly disagree) to 7 (strongly agree) beside each statement. Experts were also given the opportunity to provide comments and suggest additional items that may not have been included when developing the initial list of statements.

Consensus was defined as at SD ≤ 1.0 [11]. Statements meeting consensus were removed from the next round, while those not meeting consensus were re-proposed to the panelists for round 2.

### Delphi round 2

The list of statements that did not reach consensus from round 1 was proposed for an additional round to all 28 members. In round 2, the experts used the same voting method as described for round 1. However, feedback in the form of a statistical representation of the group scores and comments was provided in way to reduce the range of responses while preserving the anonymity of each participant. Final responses were analyzed as described for round 1 while calculation on the % of agreement (agree/strongly agree) among the group was verified as well.

### Data analysis

Data analysis was performed using Stat59. As there are no rigidly defined published standards of how to measure consensus and ranking for Delphi studies, for this study, the criteria for consensus was set a-priori as a standard deviation of less than or equal to 1.0 [12]. Statements that reached consensus were then ranked by their mean scores. Standard deviation and mean were chosen over intra-quartile range for four reasons. Firstly, Seven-point unanchored linear numeric scales are considered by many authors to be robust to the assumption of normality [13]. Secondly, for numbers of experts near 30, the underlying sampling distribution of the responses should be near normal as per the central limit theorem [14]. Thirdly, as the overall measurement of dispersion is to ensure consensus, standard deviation is more sensitive to outliers. Fourthly, as only those statements reaching consensus (with a narrow standard deviation of less than or equal to 1) it is unlikely that there will be a significant difference between mean and median in these cases. And, finally, as the Delphi studies represent a type of hybrid between qualitative and quantitative studies, it is unlikely that choice of parametric or non-parametric studies is unlikely to make a mean.

Additionally, eighty percent (80%) of participants agreeing/strongly agreeing was considered as another appropriate measure of content validity and consensus as per previous Delphi studies [15]. Comments and recommendations provided by the FGD and during round 1 were used to analyze the findings of the study.

## Results

All the twenty-eight (28) experts invited to participate in this Delphi study completed both round 1 and round 2. The experts had at least two of the criteria for inclusion in the study with the majority having sector expertise and experience in trauma and emergency care and humanitarian operations or military interventions. Broader agreement on the proposed 5 categories as key elements of the proposed conceptual framework.

### Focus group discussions

This exercise built on the consensus of the need for the TSP approach and helped to identify the five main categories forming part of a conceptual framework that contribute and support the decision to implement or not the TSP.

**Fig. 2:**
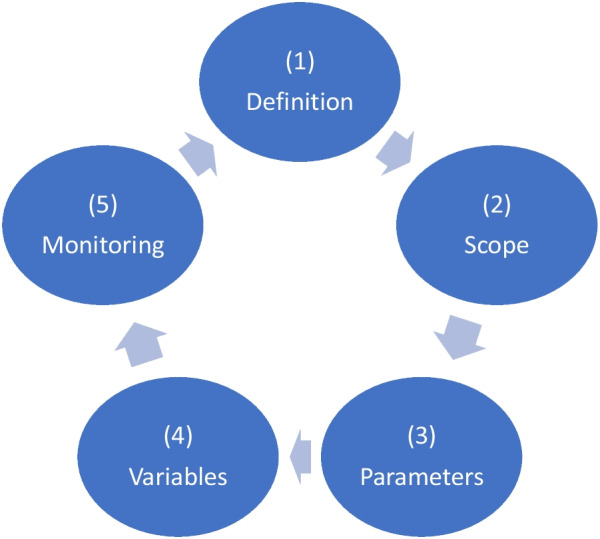
Conceptual Framework

The five main categories include the definition, scope, parameter, or characteristic that helps to define or classify the TSP, variable or external factor that should be taken into consideration in the planning and set up of the TSP, its monitoring and quality improvement. Lastly, 21 statements were developed to provide elements to support the decision-making process (Fig. 2).

### Delphi round 1

After round 1 voting was completed, 12 of 21 statements reached consensus. Looking to the results by group, the category “definition” had 2 of 4 statements reached agreement, the category “scope” 1 of 4, the category “parameter” 3 of 4, the category “variable” 2 of 5 while the category “Monitoring/Quality improvement” 4 of 4. Additionally, 24 comments were recorded many of which at operational and tactical level suggesting practices derived from personal experience and measures oriented toward concrete resources and requirements needed. 9 Statements with higher variation than 1 were re-proposed to the panelists for round 2 (Table 1).

**Table 1:** List of statements that did meet consensus after round 1

Statement	Mean	SD	Consensus
DEFINITION: TSP must be capable of functioning in resource-constrained environments	6.6	0.7	Attained
DEFINITION: The primary function of the TSP is to provide far-forward resuscitation and initial stabilization in the form of airway, hemorrhage and fracture control	6	0.9	Attained
SCOPE: The TSP has an important triage role, rapidly transferring the more serious injuries to a higher level of care and identifying minor injuries	6.5	0.9	Attained
PARAMETER: Mobility and/or Flexibility—The TSP should ensure an agile system to move and/or expand medical services based on the changing needs	6.4	1.0	Attained
PARAMETER: Transport—Adequate transport and transfer to a receiving facility that has the capability to provide more advanced care should be available	6.6	0.6	Attained
PARAMETER: Safety—The TSP should be an environment of care that is safe for patients and health care personnel with risk management plans that are context and area-specific	6.7	0.5	Attained
VARIABLE: Chain of referral—There should be a level of integration across the chain of care with continuity from POI to definitive treatment and rehabilitation	6.6	0.7	Attained
VARIABLE: Conflict dynamics—Combat strategies, intensity, and geographical location may influence clinical presentations at TSP	6.4	0.8	Attained
MONITORING/QUALITY IMPROVEMENT: Indicators and metrics which monitor areas where TSP can make a difference in patient outcomes or staff safety should be developed	6.7	0.5	Attained
MONITORING/QUALITY IMPROVEMENT: Time per patient spent at the TSP should be carefully monitored although its interpretation is dependent on several factors	6.1	0.8	Attained
MONITORING/QUALITY IMPROVEMENT: Transport time to a higher level of care should be carefully monitored although its interpretation is dependent on several factors	6.4	0.7	Attained
MONITORING/QUALITY IMPROVEMENT: Mechanism of injury and anatomic injury patterns are important criteria in monitoring and predicting workload and should be tracked	6.4	1.0	Attained

### Delphi round 2

**Fig. 3:**
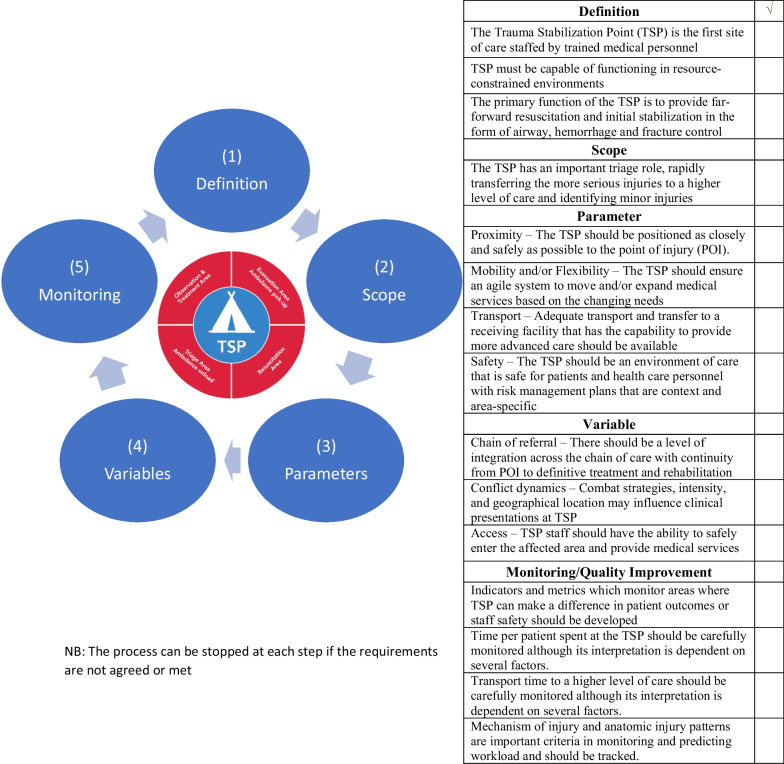
Proposed conceptual framework

After round 2 voting was completed, panel members reached consensus on 1 statement under the category “definition”. Despite a SD slightly above 1, the statement under the category “parameter” such as “Proximity—The TSP should be positioned as closely and safely as possible to the point of injury (POI)” was included under the final approved list having met 89% agreement. The same approach was used for the statement under the category “variable”: “Access—TSP staff should have the ability to safely enter the affected area and provide medical services” having met 82% agreement. A total of 6 statements did not reach consensus (Tables 2, 3 and Fig. 3).

**Table 2:** List of statements that did meet consensus after round 2

Statement	Mean	SD	Consensus
DEFINITION: The Trauma Stabilization Point (TSP) is the first site of care staffed by trained medical personnel	6	0.8	Attained
PARAMETER: Proximity—The TSP should be positioned as closely and safely as possible to the point of injury (POI)	6.4	1.2	Not attained (adjusted—89% agreement)
VARIABLE: Access—TSP staff should have the ability to safely enter the affected area and provide medical services	6.2	1.4	Not attained (adjusted—82% agreement)

**Table 3:** List of statements that did not meet consensus

Statement	SD	Consensus
DEFINITION: The utility of the TSP should be considered in all conflict settings even where fighting is sporadic or frontlines are poorly defined	1.6	Not attained
SCOPE: The TSP must be prepared to initially manage acute medical conditions in addition to trauma	1.5	Not attained
SCOPE: Uncomplicated minor injuries can be managed at the TSP	1.9	Not attained
SCOPE: Penetrating and blast injuries should (always) be considered for referral to higher level of care	1.2	Not attained
VARIABLE: Civil-military coordination – Interaction with the military and other armed groups must be established. This relationship is dependent on the medical needs and the roles and responsibilities of the military and other armed groups	1.5	Not attained
VARIABLE: Medical expertise – Capacity and capability of the medical staff should vary within the trauma care system in context with the local health care delivery system	1.1	Not attained

## Discussion

This study describes the development of a consensus-based framework to support the decision to implement frontline care services. This framework presents five main categories identified as interconnected components that facilitate the decision to implement or not the TSP. Under each component, several elements have been identified as being able to guide the decision to utilize such capability in high-risk security and resource constrained settings. The TSP has the potential to prevent death and disability, careful analysis of the findings of the study per category is presented.

### Definition

The application of military standards of trauma care closed to the frontline has challenged existing humanitarian principles and some humanitarian organization’s modus operandi [16]. The use of a TSP is not considered appropriate for all conflict settings in particular where fighting is sporadic or frontlines are poorly defined. This is in contrast to the vision of the military medical community challenged by the nonlinear battlefields of Iraq, Afghanistan and the Arabian Peninsula, calling for a revision of the doctrine, training and the concept of the Role 1 care (tactical combat casualty care) [17].

### Scope

The implementation of the TSP model during the Gaza trauma response has been shown to save lives and decrease the burden on already overwhelmed referral hospitals [18, 19]. It is envisaged that tools and lessons learnt can be adapted and applied to countries that are experiencing conflict and civil unrest in the region [18]. The scope of the TSPs was set around two main roles: the triage, treatment and discharge of patients with minor injuries and the triage, stabilization and referral of critical patients with life-threatening or limb-threatening injuries. A similar approach (First Aid Post) was applied in Afghanistan recognizing a more variable pattern of injuries and lengthy delay from injury to hospital treatment for civilians [20]. The perspective from the panel members was prudence in relation to the scope of the TSP and the risk of a broader (and inappropriate) spectrum of procedures performed at the site. This suggests the need to invest in a wider awareness and clarification of the scope of the TSP, with consideration of recent available guidance from the World Health Organization [21].

### Parameters

The approach applied in both combat and civilian emergency medical systems (EMS) considers the proximity to, the provision of lifesaving interventions at the site of illness or POI and reducing time to definitive medical care. Recognizing dissimilarity between combat and civilian trauma care, similar factors appear to affect the implementation of the TSP such as resource limitations, extreme environments, varying evacuation time and transportation platforms [21, 22]. Safety of patients and health care personnel is a paramount and risk management plans must be context and area-specific [23]. TSPs would ideally be located within the “platinum 10 min” of the POI or within 20 min considering the contextual constraints [9, 21]. However, others will argue that evidence for operational decisions based only on the Golden Hour of Trauma is weak and time to treatment should not be over-emphasized [24]. A short transport time is still worth pursuing, best achieved through robust evacuation resources and processes. All these factors should be taken into account when planning the trauma care pathway, including the location of the TSPs, a trauma by-pass system and the fact that in many situations aeromedical evacuation for civilians is not an option [25, 26].

### Variables

The scope and level of interaction between civilian and military forces and other armed actors is always context-dependent involving the adoption of relevant measures to minimize the risk to civilians and the analysis of how to operate effectively. However, regardless of the situation, the type and scale of threats to civilians must be identified, including who is vulnerable and why, to establish the basis for intervention. Existing medical capacity and capability and their implications for trauma system organization must be identified [27, 28]. Variation in the responses of the panel members and strong emphasis on humanitarian principles suggest the need for continue debate on this important variable.

### Monitoring

There was group consensus regarding the need for a monitoring and a quality improvement system. TSP and hospital data help to periodically assess the adequacy of the chain of casualty care, the efficiency of first-aid measures and the evacuation system itself. Hospital mortality decreases as evacuation time increases; thus rapid evacuation is of vital importance [24, 29]. Therefore, time per patient spent at the TSP and transport time to higher level of care should be carefully monitored although their interpretation is dependent on several factors. For example, prehospital professionals are generally expect to keep trauma scene-time stabilization under 10 min and report fatality rates during transportation. Similarly, although recognizing the quality of emergency and trauma care in resource-constrained settings is understudied, potential improvements could be made by analyzing and periodically reassessing the existing transport patterns and targeting cost-effective outreach of trauma care [30].

Several considerations have been made by the group of experts towards the importance of defining the TSP functionality. Of relevance, the definition of the minimum requirements in terms of skills, staff, equipment and resources needed as well as a curriculum for civilian medical systems and personnel to operate clinically in semi-permissive and non-permissive environments.

### Strengths and limitations

A multidisciplinary panel of physicians, nurses, paramedics, humanitarian and military experts, and policy makers from over 17 countries came to a consensus on this conceptual framework. The rigorous Delphi technique enabled statements to be honed and its anonymity reduced the effects of dominant individuals; often a concern within group-based processes that collect and synthesize information. However, the process may have benefited from a face-to-face meeting at its end phase to allow experts to exchange important information and clarify their reasons for disagreements. Particularly, it seems that the different profiles and experiences of the experts played a key role in the deviation observed in some statements due to their professional position/vision of the problem. A possible face-to-face meeting was not included in the proposed methodology and authors decided to respect the anonymity agreed at the beginning of the process.

## Conclusion

Using consensus-based evidence, this study presents a conceptual 5-step framework to support the decision to implement or not the TSP. Recognizing lack of empirical data on the benefit of such intervention, the proposed framework and categories add a structure to the decision-making process. Following the steps from 1 to 5 will allow for better definition of role, extent, and scale of such intervention. A very important step to support emergency and trauma care planning and implementation efforts. Additional research and debate are required to clearly define the scope, its functionality and the integration of such approach in asymmetric and urban warfare for the benefit of populations in conflict.

## Data Availability

The data that support the findings of this study were generated from the Stat59 online platform. However, restrictions apply to the availability of these data due to the nature of the Delphi study, requiring the ability to associate responses with the individual participants, and so are not publicly available. Data are however available from the authors upon reasonable request.
